# Balancing Work and Life: A Conversation with Fiona Watt

**DOI:** 10.1002/stem.53

**Published:** 2009-04

**Authors:** Majlinda Lako, Susan Daher

## About Dr. Watt

Dr. Fiona Watt received a B.A. in Natural Sciences in 1976 and an M.A. in 1979 from Cambridge University, U.K. She obtained her D.Phil. in 1979 from the Sir William Dunn School of Pathology, University of Oxford, for her thesis “Microtubule-organizing centers in cells in culture and in hybrids derived from them.” After a 2-year postdoctoral fellowship with Dr. Howard Green at the Massachusetts Institute of Technology, she took a position as Head of the Molecular Cell Biology Laboratory, Kennedy Institute of Rheumatology, in London. In 1987 she moved to the Cancer Research U.K. London Research Institute, where she was the Head of the Keratinocyte Laboratory until 2006. She is currently the Deputy Director for both the Cancer Research U.K. Cambridge Research Institute and the Wellcome Trust Centre for Stem Cell Research, and the Herchel Smith Professor of Molecular Genetics at Cambridge University.

Her lab is interested in how the proliferation, differentiation, and tissue assembly of epidermal stem cells and their progeny are controlled, and how these processes are perturbed in cancer, using mammalian epidermis as a model system. Some of her current projects are concerned with self-renewal and lineage selection by human and mouse epidermal stem cells, the role of stem cells in epidermal tumor formation, and the assembly and function of the epidermal cornified envelope.

Dr. Watt has organized numerous prestigious international stem cell meetings. She is currently co-organizing, with Dr. Shinya Yamanaka, the Keystone Symposium on Stem Cells to be held in Colorado in 2010. Her many awards and honors include the American Society for Cell Biology Women in Cell Biology Senior Award, 2008; Honorary Foreign Member, American Academy of Arts and Sciences, 2008; and President, International Society for Stem Cell Research, 2008. She serves on several advisory boards, including the Scientific Advisory Board, Medical Research Council Clinical Sciences Centre, the International Scientific Advisory Board of the Wellcome Trust Centre for Gene Regulation and Expression, the North East England Stem Cell Institute Scientific Advisory Board, and the American Society for Cell Biology Council. She is actively involved in communicating science to the public, by participating in numerous lectures and interviews, as well as television programs and podcasts. She has been the Editor-In-Chief of the *Journal of Cell Science* since 1992.

### Being a Scientist Is “Hardwired”

Fiona Watt knew from the time she was a young girl that she wanted to be a scientist; she had her own lab coat and was always playing with chemistry sets and keeping pet frogs. “I think that being a scientist is in a sense hardwired, and there are people who just couldn't conceive of being anything else” she explains. And now, that is the type of person she looks for to mentor in her lab. “I want people who are really motivated. I'm very happy if they've done something different before they've made a commitment to science, for example, somebody who did a first degree in English literature and then switched fields. But I want to know when they join my lab that they're really serious. If they're serious then I will support them to the best of my ability. I want to make sure that they have a project that is identifiably theirs, where they can see a paper on the horizon, but I also want them collaborating with other people in the lab so that we make more progress by helping one another.”

Dr. Watt's own path to her scientific career has been quite unlike what most young scientists would experience today. “As a Ph.D. student my project was to show if tumor cells had microtubules. The technique of immunofluorescence microscopy was really just in its infancy. My boss asked some investigators in Germany for an antibody to tubulin that we could use, and they said well, if you send your student to us we'll do the experiments together. So after 2 weeks working in Germany I had obtained the answer to my Ph.D. project! My boss said ‘Well that's good, now you can amuse yourself for the next 2 years.’ Of course you would never do that now, because the lab heads need to get publications. But that experience made me very independent, and I did have a great time. But this also left me feeling rather undereducated, because nowadays my students learn and have access to many different techniques, but my training was more one dimensional.” Then, after a short post doc of only 2 years in the U.S., Dr. Watt was hired in an arthritis institute in the U.K. “I do feel that my time from being a Ph.D. student to running a lab was so short that I didn't have as much chance to be at the bench myself as I would have liked, and I really enjoy bench science. So looking back, maybe I should not have been in such a hurry.”

### “I Feel Very Optimistic for Young Stem Cell Scientists Right Now That They'll Get a Good Job and Be Able to Fund Their Research”

One difficulty that Dr. Watt faced as a post doc and new investigator was that the field of stem cells was relatively new and undeveloped. “When I was first interested in stem cells they really weren't very respectable. Developmental biology dominated this area, and developmental biologists just couldn't understand why you would be interested in what was happening in adult tissues. So in a sense it's easier for young scientists now because we have a genuine, scientifically rigorous field. But the definition of a ‘young scientist’ is certainly changing. I recently saw a report out in the U.S. that the average age for getting a first RO1 NIH grant is over 40. It all just seems to take longer today to establish an independent lab.

As for whether or not it's easy to get a job, the job market for stem cell research is really buoyant at the moment. And I think at least in Britain the climate for getting an independent job is much better than it was 20 years ago. That may change and become more difficult in the future because of the current economic downturn. But I'm very upbeat and optimistic for the scientists I'm producing at the moment, that they will get good jobs, and that they'll be able to fund their research without too much of a struggle. It's always hard to get funding for your research, but I think everything looks very good for them at the moment.”

Even in this buoyant job market, Dr. Watt puts a great deal of time and effort into preparing her students for interviews, and helping them land their first job. “I really put a lot of effort into helping them with their first fellowship proposal, and practicing their interview, things like that. Those are skills that can make or break you. You need to be able to explain what your vision is for your lab; that's really important. Fellowship proposals may have to be written over many times to make sure they are a plausible, and we keep practicing the interviews. I really want members of my lab to succeed. I suppose my mentorship is not so intense after they've left the lab, but I do keep in touch with them and if there's the opportunity to collaborate or provide reagents or get them invited to conferences then I can help. I like to make sure that they're visible as independent people. And it's really great to see people make it.'

### “Looking After a Lab Is Like Tending a Garden”

It is an incredibly admirable accomplishment that Dr. Watt has been able to establish herself as a leader in the field, while still maintaining a full and rich family life with a spouse and three kids. “Before my first child arrived I was working such long hours that I was beginning to get rather grumpy. And I was sure that my career would end when my son was born, but actually it was just fine. Just the shear pleasure having him around and still being able to do the science has been great. It's been very positive. I take my eldest child to a lot of conferences and so now he is well known on the Stem Cell circuit.

But I think there's really no right time to have kids. I was lucky that I already had tenure when I had kids, and I had a reasonable sized lab by that stage, so I had more control over my time. I suspect it's really difficult to have kids while you're setting up your lab. And the other strategy, of having children while you're a post doc or Ph.D. student has pros and cons as well. I would feel very sad if women scientists were somehow being robbed of their fertility because they were waiting so long to have kids when the time is right. And of course I can see how you could have a very fulfilling life without having kids. Different people lead their lives in different ways.”

“You only live once, so it's really important to enjoy the work you are doing. One talent I have is to avoid worrying about things I can't do anything about. I find it very easy, as soon as I get home, to just switch off science and enjoy being with the kids. You shouldn't worry about the kids when you're in the lab and you shouldn't worry about the lab when you're with the kids. Otherwise, you'll go nuts!

Looking after a lab is sort of like tending a garden. If you forget to water it, you can't just go back in and have it look as good as it would have been if you hadn't neglected it. That's an important lesson I've learned over the last couple of years. It's so important to define what's important to you in your work. What's important to me is my lab and the people in it and doing science that I find exciting. And I'm really feeling positive about how things are going in the field; the stem cell field is becoming mature and (reasonably) respectable, with an interdisciplinary nature and the potential to really benefit human kind.”

**Figure 1:**
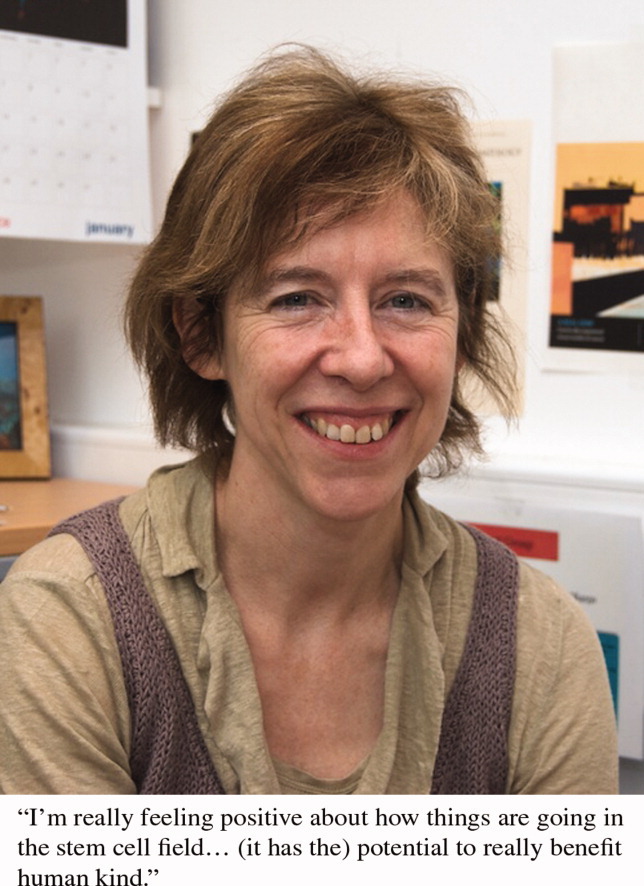
Fiona Watt, D.Phil. CR-UK Cambridge Research Institute and Wellcome Trust Centre for Stem Cell Research, Cambridge UK.

